# The epidemiology of aerobic physical activity and muscle-strengthening activity guideline adherence among 383,928 U.S. adults

**DOI:** 10.1186/s12966-019-0797-2

**Published:** 2019-04-18

**Authors:** Jason A. Bennie, Katrien De Cocker, Megan J. Teychenne, Wendy J. Brown, Stuart J. H. Biddle

**Affiliations:** 1 Physically Active Lifestyles Research Group (USQ PALs), Centre for Health, Informatics, and Economics Research, Institute for Resilient Regions, University of Southern Queensland, Education City, Springfield Central, Brisbane, QLD 4300 Australia; 2 Institute for Physical Activity and Nutrition (IPAN), School of Exercise and Nutrition Sciences, Deakin University, Building LA, 70, Elgar Rd, Burwood, VIC 3125 Australia; 3 Centre for Research on Exercise, Physical Activity and Health, School of Human Movement and Nutrition Sciences, The University of Queensland, Human Movement Studies Building, St Lucia, QLD 4067 Australia

**Keywords:** Public health, Physical activity, Resistance training, Concurrent training, Aerobic exercise

## Abstract

**Background:**

The World Health Organization’s ‘Global Recommendations on Physical Activity for Health’ state that adults should engage in regular moderate-to-vigorous intensity aerobic physical activity (MVPA; e.g. walking, running, cycling) and muscle-strengthening activity (MSA; e.g. strength/resistance training). However, assessment of both MVPA and MSA is rare in physical activity surveillance. The aim of this study is to describe the prevalence, correlates and chronic health conditions associated with meeting the combined MVPA-MSA guidelines among a population representative sample of U.S. adults.

**Methods:**

In this cross-sectional study, data were drawn from the U.S. 2015 Behavioral Risk Factor Surveillance System. During telephone interviews, MVPA and MSA were assessed using validated questionnaires. We calculated the proportions meeting both the global MVPA and MSA physical activity guidelines (MVPA ≥150 min/week and MSA ≥2 sessions/week). Poisson regressions with a robust error variance were used to assess: (i) prevalence ratios (PR) for meeting both guidelines across sociodemographic factors (e.g. age, sex, education, income, race/ethnicity); and (ii) PRs of 12 common chronic health conditions (e.g. diabetes, coronary heart disease, hypertension, depression) across different categories of physical activity guideline adherence (met neither [reference]; MSA only; MVPA only; met both).

**Results:**

Among 383,928 adults (aged 18–80 years), 23.5% (95% CI: 20.1, 20.6%) met the combined MVPA-MSA guidelines. Those with poorer self-rated health, older adults, women, lower education/income and current smokers were less likely to meet the combined guidelines. After adjustment for covariates (e.g. age, self-rated health, income, smoking) compared with meeting neither guidelines, MSA only and MVPA only, meeting the combined MVPA-MSA guidelines was associated with the lowest PRs for all health conditions (APR range: 0.44–0.76), and the clustering of ≥6 chronic health conditions (APR = 0.33; 95% CI: 0.31–0.35).

**Conclusions:**

Eight out of ten U.S. adults do not meet the global physical activity guidelines. This study supports the need for comprehensive health promotion strategies to increase the uptake and adherence of MVPA-MSA among U.S. adults. Large-scale interventions should target specific population sub-groups including older adults, women, those with poorer health and lower education/income.

**Electronic supplementary material:**

The online version of this article (10.1186/s12966-019-0797-2) contains supplementary material, which is available to authorized users.

## Background

More than 50 years of epidemiological research demonstrates that physical inactivity is an independent risk factor for all-cause mortality and multiple chronic health conditions, including cardiovascular disease, type 2 diabetes, colon/breast cancer and depression [1–7]. Since the mid-1970s, physical activity recommendations solely focused on moderate-to-vigorous aerobic physical activity (MVPA; e.g. walking, cycling, running) [8]. However, over the past decade, global physical activity guidelines for public health have included muscle-strengthening activities (MSA; e.g. strength/resistance training) [7]. The 2010 World Health Organization’s (WHO) ‘Global Recommendations on Physical Activity for Health’ state that adults (18–64 years) should engage in: (i) 150 min/week of moderate-intensity aerobic physical activity, or 75 min/week of vigorous-intensity aerobic physical activity, or an equivalent combination of both; and (ii) two or more days per week of MSA involving major muscle groups [7].

The inclusion of MSA in physical activity recommendations is due to the scientific evidence showing that MVPA and MSA may have unique and/or cumulative health benefits [9]. MVPA is primarily associated with a reduced risk of cardiovascular disease [1, 10], diabetes [11], colon/breast cancer [12–14], depression [15, 16] and cognitive decline [17]. While comparably fewer data are available on MSA, this activity is typically associated with increased skeletal muscle strength, mass, bone density [18–22], ability to perform activities of daily living, reduced risk of falls [23, 24] and enhanced glucose/lipid metabolism [25]. Recent evidence from prospective cohort studies suggests that, compared with meeting one guideline, meeting both aerobic MVPA and MSA guidelines was associated with lower risk of all-cause mortality [26, 27]. Moreover, we have recently shown that compared to meeting one guideline, concurrent MVPA-MSA is associated with favourable cardiometabolic outcomes among a sample of ~ 10,000 Korean adults [28].

Despite being recommended globally, compared with MVPA [29, 30], few public health surveillance data are available on the epidemiology of meeting both MVPA and MSA guidelines. In the U.S. for example, estimates from the 2011 Behavioral Risk Factor Surveillance System [31] and 2016 National Health Interview Survey [32] showed that ~ 22% of adults meet both the MVPA and MSA guidelines. The lowest prevalences were in people with low education (12.0%), the obese (13.5%) and those aged over 65 years (15.9%) [31]. However, in those studies, few other sociodemographic and lifestyle factors were included and multivariable analyses were not conducted [32, 33]. Regular reporting of health behaviors by population sub-groups is essential for identifying the most at-risk population sub-groups, for mapping trends over time and for developing health policy [34].

In addition, a limitation of the available epidemiological literature on MVPA and MSA is that some people who are classified as meeting the MVPA guideline may also meet the MSA guideline, and vice versa [26, 35, 36]. To our knowledge, the associations between mutually exclusive groupings of physical activity guideline adherence (i.e. ‘meet neither’ vs. ‘meet MVPA only’ vs. ‘meet MSA only’ vs. ‘meet both’) and chronic health conditions have not been examined using publically available surveillance data. Since MSA and MVPA may have unique and/or cumulative health benefits [9], it is important to ascertain whether these two types of exercise have mutually exclusive or different associations with specific health outcomes.

The primary aim of this paper is to describe the prevalence and sociodemographic/lifestyle correlates of meeting both the MVPA and MSA guidelines in a large population sample of U.S. adults. A further aim is to report on the independent associations between different combinations of MVAP and MSA guideline adherence and chronic health conditions.

## Methods

### Sample

Data were drawn from the 2015 ‘Behavioral Risk Factor Surveillance System’ (hereafter: BRFSS 2015) [37]. Initiated in 1984 and conducted yearly, the BRFSS collects state-specific data on health risk behaviors that are relevant to public health among U.S. adults [38]. The BRFSS 2015 was approved by the National Center for Health Statistics Research Ethics Review Board. On being contacted by telephone, participants were provided with a description of the aim of the BRFFS 2015 via a standardized text. Participants were asked to provide verbal consent to agree to take part in the telephone interview. This analysis used the BRFSS 2015 de-identified public data and was considered exempt from human subjects review by the University of Southern Queensland Review Board. Detailed descriptions of the methodology and data collection processes used in the BRFSS 2015 are available elsewhere [39].

The BRFSS 2015 used both landline telephone and mobile/cell phone surveys. During the cell phone survey, interviewers collected data from one adult residing in a private residence/college housing. In the landline survey, interviewers collected data from a randomly selected adult in individual households [40]. Data were collected from the state health departments of all 50 U.S. states, the District of Columbia, Puerto Rico, and Guam [39]. The median response rate for the combined landline and cell phone was 47.2% (range: minimum = 33.9% in California; to maximum = 61.1% in Utah) [39]. Initially, data were collected from 441,456 respondents. For the present analysis, participants were excluded if data were missing for MVPA or MSA (*n* = 57,528, 13.0% of the total sample). To enhance generalizability, we did not utilize any other exclusion/inclusion criteria. Moreover, since the key physical activity guidelines (i.e. dose of MVPA and MSA) apply to both adults (aged 18–64 years) and older adults (aged ≥65 years) [41], this study includes adults aged ≥18 years.

### Physical activity assessments

A detailed overview of the development of the physical activity questionnaire items used in the BRFSS is available elsewhere [42]. All aerobic MVPA and MSA estimates were calculated using previously standardized scoring protocols [43].

Self-reported MVPA was assessed by asking participants “*During the past month, other than your regular job, did you participate in any physical activities or exercises such as running, calisthenics, golf, gardening, or walking for exercise*?”. If they answered ‘yes’, they were then asked “*What type of physical activity or exercise did you spend the most time doing during the past month?”*, “*How many times per week or per month did you take part in this activity during the past month?* and “*When you took part in this activity, for how many minutes or hours did you usually keep at it?*”. BRFSS researchers coded the answers for each activity as either ‘aerobic’ or ‘non-aerobic’ using a previously developed list of 56 leisure-time sports and recreation activities [43]. In that coding protocol, examples of aerobic activities included walking, hiking, biking, swimming and running, while non-aerobic activities included gardening, painting, golf and bowling [43]. To count toward meeting the MVPA guideline, activities had to be first aerobic, and performed for ≥10 min at a time. MET values were used to classify the intensity of the activities, and were estimated using sex-specific regression equations [43]. Vigorous-intensity activity was categorized as having a MET value of at least 60% of a person’s maximal cardiorespiratory capacity. Moderate-intensity activity was defined as ≥3.0 metabolic equivalents and less than the respondent’s vigorous-intensity cut point described above. These survey items have acceptable test-retest reliability (Cohen’s kappa [*k*] = 0.67; 95% CI: 0.48, 0.88) and concurrent validity (*k* = 0.41; 95% CI: 0.17, 0.66) (using physical activity log as the standard) [42].

Self-reported MSA was assessed by asking: “*During the past month, how many times per week or per month did you do physical activities or exercises to strengthen your muscles?*”. When considering this question, respondents were prompted, “*Do not count aerobic activities like walking, running, or bicycling. Count activities using your own body weight like yoga, sit-ups or push-ups and those using weight machines, free weights, or elastic bands*” This MSA item has shown evidence of test-retest reliability (*k* = 0.85; 95% CI: 0.71, 0.99) [42], and convergent validity [36].

To assess the primary research aim, the sample was dichotomized as either: (i) meeting both the MVPA and MSA guidelines (150 min/week of moderate-intensity aerobic physical activity, or 75 min/week of vigorous-intensity aerobic physical activity, or an equivalent combination of both [hereafter MVPA] and ≥ 2 sessions/week of MSA) or (ii) not meeting both MVPA and MSA guidelines (not meeting the above classification). To assess the secondary research aim, each respondent was categorized into one of four classification categories: (i) ‘Meet neither’ (MVPA = 0–149 min/week & MSA = 0–1 sessions/week); (ii) ‘Meet MSA only’ (MSA ≥2 sessions/week & MVPA = 0–149 min/week); (iii) ‘Meet MVPA only’ (MVPA ≥150 min/week & MSA = 0–1 sessions/week); and (iv) ‘Meet both’: (MVPA ≥150 min/week & MSA ≥2 sessions/week).

### Co-variates

Sociodemographic (e.g. age, sex, income, race/ethnicity, education) and lifestyle characteristics (e.g. self-rated health, self-reported body mass index [BMI], smoking) were assessed using standardized questionnaire items [44] and were included as co-variates due to their established association with participation in physical activity [45]. For self-rated health, respondents were asked “*Would you say that in general your health is*: (i) ‘*Excellent*’; (ii) ‘*Very good*’; (iii) ‘*Good*’; (iv) 4 ‘*Fair*’; (v) 5 ‘*Poor*’. Body Mass Index (BMI) was calculated from self-report height and weight and categorized as: (i) < 18.5 kg/m^2^ (*underweight*); (ii) from ≥18.5 kg/m^2^ to < 25 kg/m^2^(*acceptable weight range*); (iii) from ≥25 kg/m^2^ to < 30 kg/m^2^ (*overweight*); and (iv) ≥30 kg/m^2^ (*obese*). To assess smoking status, four groups were created: (i) ‘*never smoked*’; (ii) ‘*former smoker*’; (iii) ‘*current smoker (some days)*’; and (iv) ‘*current smoker (daily)*’.

### Chronic health conditions

Twelve chronic health conditions were assessed, including six cardiovascular-related chronic conditions (hypertension, high cholesterol, myocardial infarction, coronary heart disease, stroke and diabetes); and six general chronic conditions (depressive disorder, chronic obstructive pulmonary disease [COPD], asthma, kidney disease, cancer [non-skin] and arthritis/rheumatoid arthritis). These chronic health conditions are globally prevalent and associated with significant morbidity and mortality [46]. To assess each condition, respondents were asked “*Has a doctor, nurse or other health professionals ever told you that you had any of the following?*”. The three response options: (i) ‘yes’; (ii) ‘no’; or (iii) ‘don’t know/unsure’, were collapsed into: (i) ‘yes’ or (ii) ‘no’ (collapsing ‘no’ and ‘don’t know/unsure’). As in previous studies, both individual and total number of general chronic health conditions (range: 0 to ≥6) are reported [28, 47–49]. See Additional file 1 for a description of the unadjusted prevalence of individual and total chronic diseases across each category of physical activity guideline adherence.

## Statistical analysis

Analyses were conducted using the Complex Samples module of SPSS version 23 (SPSS Inc. an IBM Company, Chicago, IL, USA). Data were weighted to provide population estimates that accurately represent the U.S. population across sociodemographic groups (age, sex, education, income). Each BRFSS 2015 respondent was provided with an individual stratum weight, which was used to correct for non-response. More detailed information on the weighting of the BRFSS 2015 sample can be found elsewhere [50].

To examine the first primary aim, weighted percentages (%) and their 95% confidence intervals (95% CI) were calculated for meeting both the MVPA and MSA recommendations, for the total sample and by sociodemographic and lifestyle characteristics. Weighted percentages (%; 95% CIs) were also calculated for ‘meeting neither’, ‘meeting MSA only’ and ‘meeting MVPA only’ for the total sample. To examine the second primary aim, Poisson regression analyses, with robust error variance, were used to calculate prevalence ratios (PRs) for meeting both the MVPA and MSA recommendations (yes/no) (dependent variable), by sociodemographic and lifestyle-related characteristics (explanatory variables). The reference groups for these analyses are shown in Table 1.

**Table 1:** Proportions^a^ meeting MVPA-MSA^b^ guidelines and adjusted prevalence ratios^c^ (APR)^d^ for meeting both guidelines

	n	Met both aerobic MVPA-MSA guidelines^b^	
		%^a^ (95% CI)	APR^c^ (95% CI)
Total	383,928	20.3 (20.1, 20.6)	–
Sex	%^a^ (n)		
Male	48.5 (162,252)	22.9 (22.5, 23.3)	1 (reference)
Female	51.5 (221,673)	18.0 (17.6, 18.3)	0.85 (0.84, 0.86)
Age			
18–24	12.5 (20,516)	29.7 (28.7, 30.8)	1 (reference)
25–34	17.1 (36,500)	23.3 (22.6, 24.0)	0.78 (0.75, 0.81)
35–44	16.3 (44,203)	19.9 (19.2, 20.6)	0.68 (0.66, 0.71)
45–54	17.4 (62,629)	18.5 (17.9, 19.0)	0.64 (0.62, 0.66)
55–64	16.8 (86,515)	16.8 (16.4, 17.3)	0.59 (0.57, 0.61)
65–74	11.7 (78,429)	17.8 (17.3, 18.4)	0.61 (0.59, 0.63)
> 75	8.1 (55,316)	15.4 (14.8, 16.1)	0.53 (0.51, 0.56)
Race/Ethnicity			
White, non-Hispanic	65.1 (296,618)	20.9 (20.6, 21.2)	1 (reference)
Black, non-Hispanic	11.0 (28,163)	20.3 (19.4, 21.1)	0.88 (0.85, 0.91)
Other race, Non-Hispanic	6.3 (163,39)	21.3 (20.0, 22.7)	1.02 (0.99, 1.06)
Multiracial, Non-Hispanic	1.4 (7150)	24.2 (22.1, 26.4)	1.12 (1.06, 1.18)
Hispanic	16.4 (30,599)	17.6 (16.9, 18.4)	0.85 (0.82, 0.88)
Employment status			
Student	5.8 (10,119)	31.5 (30.0, 33.0)	1 (reference)
Employed	56.8 (189,429)	21.8 (21.5, 22.2)	0.70 (0.67, 0.73)
Unemployed	5.7 (15,782)	19.2 (18.0, 20.4)	0.59 (0.56, 0.63)
Homemaker	6.8 (23,510)	16.1 (15.2, 17.0)	0.60 (0.56, 0.63)
Retired	18.1 (115,612)	18.3 (17.9, 18.8)	0.62 (0.60, 0.65)
Unable to work	6.8 (27,391)	9.1 (8.5, 9.8)	0.31 (0.29, 0.32)
Education level			
Graduated College	27.2 (144,604)	26.8 (26.3, 27.2)	1 (reference)
Attended College/Technical	31.5 (105,888)	21.5 (21.1, 22.0)	0.73 (0.72, 0.74)
Graduated High School	27.6 (104,353)	16.8 (16.4, 17.3)	0.54 (0.53, 0.55)
Did not graduate High School	13.7 (28,192)	12.1 (11.4, 12.9)	0.37 (0.36, 0.39)
Income (annual)			
$50,000 or more	48.3 (158,855)	20.7 (20.4, 20.9)	1 (reference)
$35,000–$50,000	13.7 (46,879)	20.0 (19.3, 20.8)	0.73 (0.72, 0.75)
$25,000–$35,000	10.4 (34,900)	17.6 (16.8, 18.5)	0.66 (0.65, 0.68)
$15,000–$25,000	16.5 (51,555)	15.7 (15.1, 16.4)	0.58 (0.56, 0.59)
Less than $15,000	11.1 (32,870)	13.3 (12.5, 14.0)	0.49 (0.47, 0.50)
Body mass index (kg/m^2^)			
Underweight (< 18.5)	1.8 (5806)	18.4 (16.5, 20.5)	0.71 (0.66, 0.76)
Acceptable weight (18.5–25)	33.4 (116,291)	26.2 (25.7, 26.7)	1 (reference)
Overweight [25–30]	35.8 (131,431)	21.3 (20.8, 21.7)	0.79 (0.77, 0.80)
Obese (≥30)	29.1 (108,271)	14.3 (13.8, 14.7)	0.50 (0.49, 0.51)
Self-rated health			
Excellent	18.7 (66,224)	32.7 (32.0, 33.4)	1 (reference)
Very good	32.5 (128,333)	23.5 (23.0, 24.0)	0.69 (0.68, 0.70)
Good	31.1 (117,639)	15.9 (15.5, 16.3)	0.47 (0.46, 0.48)
Fair	13.1 (50,936)	10.5 (9.9, 11.0)	0.31 (0.29, 0.32)
Poor	4.6 (19,917)	6.6 (6.0, 7.7)	0.21 (0.20, 0.23)
Smoking status			
Never smoked	58.7 (216,025)	22.0 (21.7, 22.4)	1 (reference)
Former smoker	24.8 (110,996)	19.9 (19.4, 20.4)	0.91 (0.89, 0.92)
Current (some days)	5.1 (15,935)	19.2 (18.1, 20.4)	0.84 (0.80, 0.87)
Current (daily)	11.4 (38,969)	13.2 (12.6, 13.9)	0.52 (0.50, 0.54)

^a^Data weighted using stratum weight provided by the Centers for Disease Control and Prevention (CDC) [50]

^b^Meeting both guidelines defined as aerobic MVPA = ≥150 min/week & MSA = ≥2 sessions/week

^c^Prevalence ratio calculated using Poisson regression with a robust error variance

^d^Adjusted for all other explanatory variables in the table

To examine the secondary aim, Poisson regressions with a robust error variance were used to calculate PRs for individual chronic health conditions (yes/no) (dependent variable) across the four physical activity guideline adherence classification categories (explanatory variables). For these analyses, not meeting either the MVPA or MSA guidelines (‘meet neither’) was used as the reference group. Similarly, this analysis was performed for the presence of 4, 5 or ≥ 6 total health conditions. Prior to conducting our final analytical models, we assessed collinearity among covariates using tests for variance inflation factor (VIF), with a VIF ≥ 2 indicating multicollinearity [51]. The VIFs ranged from 1.03–1.68, indicating no evidence of collinearity. In addition, we tested for independence of observations to safeguard that all data where appropriately fitted in the final models (i.e. no evidence of over or under dispersion).

In addition, we conducted a series of sensitivity analyses. First, with regression models unadjusted and adjusted for sociodemographic and lifestyle-related factors; and second, a sex-stratified analysis. As shown in Additional file 2, PRs were similar when models were unadjusted and adjusted for sociodemographic and lifestyle-related characteristics. As presented in Additional file 3, the sex-stratified analysis indicated the PRs were generally concordant across physical activity guideline adherence categories for men and women. Therefore, in this paper, we will present the adjusted prevalence ratios (APRs) for the reporting of chronic health conditions across categories of physical activity guideline adherence classification for the total sample.

## Results

### Sample description

Data from 383,928 adults aged 18–80 years were included in the analysis. Socioeconomic and lifestyle characteristics of participants are shown in Table 1. The majority were middle-aged (aged 35–64 years), employed, and either white or Hispanic. About one quarter had an acceptable BMI (18.5–25 kg/m^2^), and just over half had never smoked. Around one third had excellent self-rated health.

#### Physical activity guideline adherence classification categories

A total of 39.6% (95% CI: 39.3, 39.9%) ‘met neither’, 9.9% (95% CI: 9.7, 10.1%) ‘met MSA only’, 30.2% (95% CI: 30.0, 30.5%) ‘met aerobic MVPA only’ and 20.3% (95 CI: 20.1, 20.6%) ‘met both’.

### Correlates of meeting both MVPA-MSA guidelines

In the multivariate adjusted analysis, the adjusted prevalence ratio (APR) for meeting both MVPA and MSA guidelines was lower in women (APR = 0.85; 95% CI: 0.84, 0.86) than in men. APRs declined with age, but increased with education, income and self-rated health. They were lowest in those with fair and poor self-rated health (see Table 1).

Across categories of race/ethnicity, when compared with the reference group (White, non-Hispanic), Black, non-Hispanic and Hispanic adults had 15 and 12% lower APRs for meeting both MVPA and MSA guideline, respectively. In contrast, multiracial, non-Hispanic adults had higher APRs (APR =1.12; 95% CI: 1.06, 1.18) for meeting both the MVPA and MSA guidelines. APRs were lower in all employment categories than in students, and lower in all BMI categories than in healthy weight respondents. People classified as ‘obese’ were 50% less likely to meet both guidelines than healthy weight respondents. For most characteristics, the APRs followed an inverse linear gradient (see Table 1).

### Chronic health conditions by categories of physical activity guideline adherence

**Fig. 1:**
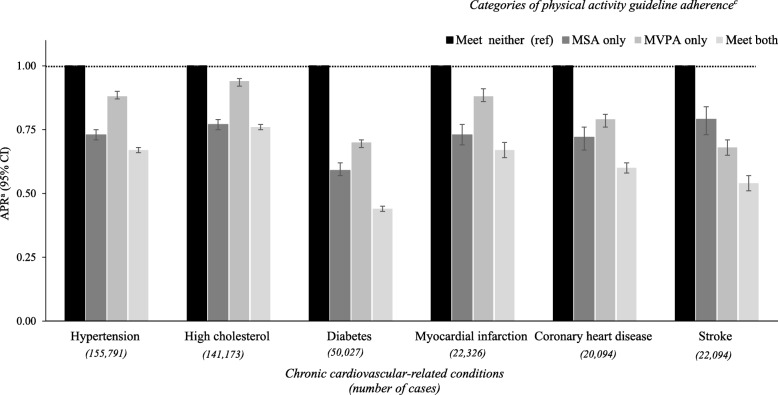
Adjusted prevalence ratios^a^ (APR; 95% CI) for cardiovascular-related conditions^b^ by categories of PA guideline adherence. ^a^Prevalence ratio calculated using Poisson regression with a robust error variance and adjusted for sex, age, race/ethnicity, employment, education, income, smoking and BMI. ^b^To be classified as having an chronic health condition a respondent had to report having a “doctor, nurse or other health professional” diagnose each condition. ^c^ Physical activity guideline adherence: ‘Meet neither’: MVPA = 0–149 & MSA = 0–1 sessions/week; ‘MSA only’; MSA = ≥2 sessions/week & MVPA = 0–149 min/week); ‘MVPA only’ MVPA = ≥150 min/week & MSA = 0–1 sessions/week; and ‘Meet both’: MVPA = ≥150 min /week & MSA = ≥2 sessions/week. (Both unadjusted and adjusted PRs are shown in Additional file 2)

APRs for chronic cardiovascular-related conditions by categories of physical activity guideline adherence are shown in Fig. 1. After adjusting for covariates, for all cardiovascular disease-related conditions, the lowest APRs were seen in those who met both guidelines (range: 0.44–0.76), followed by MSA only (0.59–0.77) and aerobic MVPA only (0.68–0.94).

**Fig. 2:**
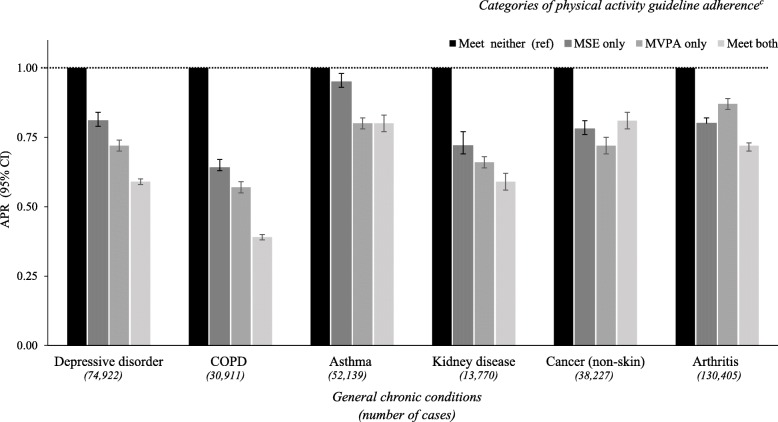
Adjusted prevalence ratios^a^ (APR; 95% CI) for general health conditions^b^ by categories of PA guideline adherence. ^a^ Prevalence ratio calculated using Poisson regression with a robust error variance and adjusted for sex, age, race/ethnicity, employment, education, income, smoking and BMI. ^b^ To be classified as having an chronic health condition a respondent had to report having a “doctor, nurse or other health professional” diagnose each condition. ^c^ Physical activity guideline adherence: ‘Meet neither’: MVPA = 0–149 & MSA = 0–1 sessions/week; ‘MSA only’; MSA = ≥2 sessions/week & MVPA = 0–149 min/week); ‘MVPA only’ MVPA = ≥150 min/week & MSA = 0–1 sessions/week; and ‘Meet both’: MVPA = ≥150 min /week & MSA = ≥2 sessions/week. (Both unadjusted and adjusted PRs are shown in Additional file 2)

**Fig. 3:**
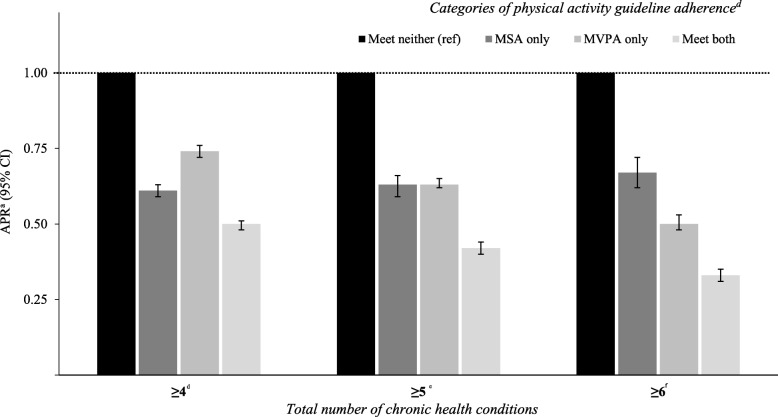
Adjusted prevalence ratios^a^ (APR; 95% CI) for total number of health conditions^b^ by categories of PA guideline adherence. ^a^Prevalence ratio calculated using Poisson regression with a robust error variance and adjusted for sex, age, race/ethnicity, employment, education, income, smoking and BMI. ^b^ To be classified as having an chronic health condition a respondent had to report having a “*doctor, nurse or other health professional*” diagnose each condition. Chronic health conditions = hypertension, high cholesterol, diabetes, myocardial infarction, coronary heart disease, stroke, depressive disorder, chronic obstructive pulmonary disease, asthma, kidney disease, cancer (non-skin) and arthritis. ^c^ Physical activity guideline adherence: ‘Meet neither’: MVPA = 0–149 & MSA = 0–1 sessions/week; ‘MSA only’; MSA = ≥2 sessions/week & MVPA = 0–149 min/week); ‘MVPA only’ MVPA = ≥150 min/week & MSA = 0–1 sessions/week; and ‘Meet both’: MVPA = ≥150 min /week & MSA = ≥2 sessions/week. ^d^reference = ≤3 adverse conditions. ^e^reference = ≤4 adverse conditions. ^f^ reference = ≤5 adverse conditions. (Both unadjusted and adjusted PRs are shown in Additional file 2)

Excluding cancer, a comparable pattern was observed for all remaining general health conditions, with the lowest APRs among those who met both guidelines for the remaining five conditions (range: 0.39–0.81) (Fig. 2). For total number of chronic health conditions, the lowest APRs were among those who met both guidelines, (range: 0.33–0.46), followed by those who met aerobic MVPA only (range: 0.50–0.60) and those who met MSA only (range: 0.67–0.69) (Fig. 3).

## Discussion

This is the first study to comprehensively assess the prevalence, correlates and health conditions associated with meeting both MVPA and MSA guidelines among a large sample of U.S. adults. The key finding was that most U.S. adults do not meet the global physical activity guidelines. A further key finding was that, compared with other physical activity categories, meeting both MVPA and MSA guidelines was independently associated with the lowest prevalence ratios for several globally prevalent chronic health conditions, including coronary heart disease, diabetes, hypertension and depressive disorder.

A recent study showed that after pooling adult aerobic MVPA levels from large population surveys, the prevalence of insufficient MVPA among U.S. adults was ~ 40% [29]. However, the present study suggests that when estimating the prevalence of combined MSA and MVPA guideline adherence, the prevalence of insufficient physical activity is twofold greater (80.7%). Indeed, the inactivity prevalence levels presented here suggest that inactivity estimates solely based on insufficient aerobic MVPA may not provide an accurate assessment of this important modifiable chronic disease risk factor at the population level.

The proportion of U.S. adults meeting both MVPA and MSA guidelines in the present study is similar to that from an analysis of the BRFSS 2011 (2011 = 20.6% vs. 2015 = 20.3%) [31]. These data suggest that MVPA-MSA levels have essentially remained unchanged over this four-year period. Similar prevalence levels have been shown in the U.K. [52], while smaller proportions of Australian adults meet both guidelines (15.0%) [53]. The present study expands on the BRFSS 2011 analysis because we assessed a larger number of sociodemographic and lifestyle factors and conducted a multivariable analysis [31]. Generally consistent with the limited studies in this area, sub-groups with lower odds of meeting both guidelines included those with poorer self-rated health, people with low education and income, women, the obese/overweight, older adults, and current smokers [33, 53].

The high prevalence of physical inactivity observed in the present study emphasizes the need for immediate public health attention to address insufficient MVPA-MSA among U.S. adults.

If government health departments expect meaningful changes in population levels of MVPA and MSA, there is a necessity for simultaneous and multilevel physical activity promotion interventions. This approach is consistent with the framework for tobacco control [54], which highlights the need for health promoting social/cultural, policy and physical environments [54]. Indeed, when compared with the U.S. prevalence estimates of smoking (15.5%) and heavy alcohol consumption (5.0%) [55], insufficient MVPA-MSA is significantly more prevalent (80.7%), hence suggesting that equal (or greater) public health attention should be orientated towards increasing population level engagement in both MVPA-MSA. However, from a physical activity promotion perspective, it is recognized that MVPA and MSA are complex behaviors, each with multiple and somewhat different levels of influence. Much research has investigated the influences on MVPA, which include factors such as exercise intention/motivation and social/physical environmental support (e.g. peer support, access to recreation facilities) [45]. Yet less is known about the influences on MSA, which are likely to be an even more complex. For example, safe/optimal MSA progression requires basic equipment (dumbbells, handheld weights,), motor skill proficiency/self-efficacy [56] and understanding of specific terms (e.g. ‘sets’ ‘repetitions’) [57].

A novel aspect of the present study is the examination of the associations between mutually exclusive groupings of physical activity guideline adherence with multiple chronic health conditions. Given the cross-sectional design of the present study, we urge caution in drawing causal inferences from these results. Nevertheless, to our knowledge, this is the first analysis of this kind among a large population sample. Overall, when compared with other categories, meeting both guidelines was associated with the lowest prevalence for globally prevalent chronic health conditions and total number of chronic conditions. These findings are consistent with recent longitudinal evidence showing that combining MVPA and MSA leads to a reduced risk of incident type 2 diabetes among ~ 100,000 U.S. women [58], independent of age, diet quality, smoking and alcohol consumption. Moreover, meeting both guidelines lead to a reduced independent risk of all-cause mortality among ~ 80,000 U.K. adults [26] and among ~ 14,000 U.S. 3+ year cancer survivors [27]. A limitation of those cohort studies is that they typically include predominately White American/European samples with higher education and income levels than the general population [26, 27, 58]. Future prospective studies with more representative samples and repeated MSA and MVPA assessments are needed to further describe the associations between these combined behaviors and health.

Within the context of the present study, our MVPA-MSA analysis of correlates was limited to sociodemographic and lifestyle factors. Similar to the extensive literature on MVPA correlates [30, 45, 59], further research is now required to assess a greater range of factors influencing engagement in MVPA and MSA concurrently. This should include examining psychosocial (attitudes, intentions, self-efficacy etc.), social (social norms, support from friends/peers etc.) and physical environmental factors (recreational facilities, neighborhood design etc.) [60]. Rather than just examining MVPA or MSA in isolation [29, 45, 56, 61], we call for future physical activity correlates research to investigate the key influences associated with engaging in these two key health behaviors concomitantly.

### Strengths and limitations

Strengths include the use of a large representative sample of U.S. adults. Moreover, the use of standardized recruitment, data collection and reduction procedures make it possible to compare our findings to similar studies and future BRFSS data. A further strength was the use of mutually exclusive groupings of physical activity guideline adherence to examine the associations with multiple chronic health conditions.

A key limitation of the present study is the use of self-report assessments of MVPA and MSA, which may have resulted in recall bias (e.g. under/over reporting, social desirability) [62]. To address these limitations, the use of device-based assessments, such as accelerometers, might have enhanced the validity of MVPA estimates [62]. For MSA, however, there is presently no alternate method to self-report assessments, and these self-reports are routinely used in physical activity surveillance [33, 52, 53]. The fact that 13.5% of the sample did not report on their MVPA-MSA levels is likely to have influenced our prevalence estimates. It is likely that those who did not report their physical activity levels are among the most physical inactive participants. Therefore, the MVPA-MSA estimates presented in this paper are potentially conservative. The cross-sectional nature of the study limits inferences of causality for the assessed health-related outcomes. Future longitudinal studies that include assessments of both MVPA and MSA are needed to establish the temporal associations between MVPA-MSA guideline adherence with health-related outcomes.

## Conclusion

Approximately 80% of U.S. adults do not meet the combined MVPA and MSA guidelines, despite documented favorable health outcomes. Future health promotion strategies to support the uptake/adherence of both MVPA and MSA among U.S. populations should target those with poorer health, older adults, women, those with low education, low income, the obese and current smokers. Moreover, prospective cohort studies are needed to confirm the temporal associations between MVPA-MSA and health outcomes observed in this cross-sectional study.

## Additional files

Additional file 1: Weighted^a^ prevalence (%) of chronic health conditions^b^ (individual and total) by categories of physical activity guideline adherence^c^ in the 2015 Behavioural Risk Factor Surveillance System sample (*n* = 383,928)^d^. (DOCX 18 kb)

Additional file 2: Prevalence ratios (PR) for individual adverse health conditions^a^ according to categories of physical activity guideline adherence^b^: overall. (DOCX 19 kb)

Additional file 3: Adjusted Prevalence ratios^a^ (APR) for individual adverse health conditions^b^ according to categories of physical activity guideline adherence^c^: overall and by sex. (DOCX 20 kb)
