# Supplementary material for: The epidemiology of aerobic physical activity and muscle-strengthening activity guideline adherence among 383,928 U.S. adults
Source: Int J Behav Nutr Phys Act. 2019 Apr 18;16:34. doi: 10.1186/s12966-019-0797-2 (PMC6472085; doi:10.1186/s12966-019-0797-2)
Supplement: Supplementary file 1 — Weighteda prevalence (%) of chronic health conditionsb (individual and total) by categories of physical activity guideline adherencec in the 2015 Behavioural Risk Factor Surveillance System sample (n = 383,928)d. (DOCX 18 kb) [file 12966_2019_797_MOESM1_ESM.docx]

| **Additional file 1:**  Weighted^a^ prevalence (%) of chronic health conditions^b^ (individual and total) by categories of physical activity guideline adherence^c^ in the 2015 Behavioural Risk Factor Surveillance System sample (n=383,928)^d^. | | | | | | |
| --- | --- | --- | --- | --- | --- | --- |
|  | | | **Meet neither^c^** | **Muscle strengthening only^c^** | **Aerobic MVPA only^c^** | **Meet both^c^** |
| **Individual chronic health conditions^b,e^** | | |  | | | |
|  | Cardiovascular disease-related conditions | | **Weighted^a^ %^e^ (95% CI)** | | | |
|  |  | Hypertension | 14.8 (14.6-15.0) | 2.5 (2.4-2.6) | 10.1 (9.9-10.3) | 4.9 (4.8-5.0 |
|  |  | High cholesterol | 16.4 (16.2-16.7) | 2.8 (2.7-3.0) | 11.9 (11.7-12.1) | 5.9 (5.8-6.1) |
|  |  | Diabetes | 14.5 (14.2-14.8) | 7.2 (6.7-7.7) | 10.8 (10.4-11.1) | 6.0 (5.7-6.4) |
|  |  | Myocardial infarction | 5.4 (5.2-5.6) | 3.1 (2.9-3.5) | 4.3 (4.1-4.5) | 2.7 (2.5-2.9) |
|  |  | Coronary heart disease | 5.2 (5.0-5.4) | 3.2 (2.9-3.6) | 4.1 (4.0-4.3) | 2.7 (2.6-2.9) |
|  |  | Stroke | 3.9 (3.8-4.1) | 2.6 (2.3-2.9) | 2.7 (2.5-2.9) | 1.9 (1.7-2.0) |
|  | General conditions | |  |  |  |  |
|  |  | Depressive disorder | 22.1 (21.7-22.5) | 16.7 (16.0-17.5) | 16.7 (16.3-17.1) | 13.2 (12.7-13.6) |
|  |  | Chronic obstructive pulmonary disease | 8.9 (8.6-9.1) | 5.0 (4.6-5.4) | 5.5 (5.3-5.7) | 3.5 (3.3-3.8) |
|  |  | Asthma | 15.0 (14.7-15.4) | 14.6 (13.9-15.4) | 13.0 (12.6-13.3) | 13.4 (12.9-13.9) |
|  |  | Kidney disease | 3.6 (3.5-3.8) | 2.3 (2.0-2.6) | 2.4 (2.3-2.6) | 1.6 (1.5-1.8) |
|  |  | Cancer (non-skin) | 7.3 (7.1-7.5) | 5.0 (4.7-5.5) | 7.5 (7.3-7.8) | 5.4 (5.1-5.7) |
|  |  | Arthritis/rheumatoid arthritis | 29.3 (28.9-29.8) | 20.3 (19.5-21.0) | 25.9 (25.4-26.3) | 18.7 (18.2-19.2) |
| **Total number of** **chronic health conditions^f^** | | | **Weighted^a^ %^f^ (95% CI)** | | | |
|  | 0 | | 30.2 (29.7-30.7) | 42.9 (41.9-44.0) | 32.7 (32.2-33.3) | 43.1 (42.4-43.8) |
|  | 1 | | 22.7 (22.3-23.1) | 25.2 (24.3-26.1) | 25.2 (24.7-25.7) | 26.4 (25.8-27.0) |
|  | 2 | | 16.7 (16.3-70.0) | 13.9 (13.2-14.6) | 17.9 (17.5-18.3) | 15.0 (14.5-15.5) |
|  | 3 | | 12.2 (11.9-12.5) | 7.8 (7.3-8.4) | 11.6 (11.3-11.9) | 8.3 (8.0-8.6) |
|  | 4 | | 8.1 (7.9-8.4) | 4.8 (4.4-5.3) | 6.6 (6.3-6.8) | 3.9 (3.7-4.2) |
|  | 5 | | 5.0 (4.8-5.2) | 2.6 (2.4-2.9) | 3.3 (3.1-3.5) | 1.8 (1.7-2.0) |
|  | ≥6 | | 5.1 (4.9-5.3) | 2.7 (2.5-3.0) | 2.7 (2.6-2.9) | 1.5 (1.3-1.6) |
| ^a^ Data weighted using stratum weight provided by the Centers for Disease Control and Prevention (CDC) (54).  ^b^ to be classified as having an chronic health condition a respondent had to report having a “*doctor, nurse or other health professional*” diagnose each condition.  ^c^ Physical activity guideline adherence:‘Meet neither’ defined as moderate-to-vigorous physical activity (MVPA) = 0-149 minutes/week & muscle strengthening exercise (MSE) = 0-1 sessions/week; ‘Muscle strengthening exercise only’ defined as MSE = ≥2 sessions/week & aerobic MVPA = 0-149 minutes/week; ‘MVPA only’ defined as aerobic MVPA = ≥150 minutes/week & MSE = 0-1 sessions/week; ‘Meet both’ defined as aerobic MVPA = ≥150 minutes/week & MSE = ≥2 sessions/week.  ^e^  Percentages are presented relative to the proportion of total number within each category of physical activity guideline adherence. For example, among those who meet neither guideline, 14.8% had hypertension.  ^f^ Percentages are presented for the total sample among each category of physical activity guideline adherence. For example, among those who meet neither, 30.2% had no chronic health conditions. | | | | | | |
