# Supplementary material for: The epidemiology of aerobic physical activity and muscle-strengthening activity guideline adherence among 383,928 U.S. adults
Source: Int J Behav Nutr Phys Act. 2019 Apr 18;16:34. doi: 10.1186/s12966-019-0797-2 (PMC6472085; doi:10.1186/s12966-019-0797-2)
Supplement: Supplementary file 3 — Adjusted Prevalence ratiosa (APR) for individual adverse health conditionsb according to categories of physical activity guideline adherencec: overall and by sex. (DOCX 20 kb) [file 12966_2019_797_MOESM3_ESM.docx]

| **Additional file 3:** Adjusted Prevalence ratios^a^ (APR) for individual adverse health conditions^b^ according to categories of physical activity guideline adherence^c^: overall and by sex | | | | | |
| --- | --- | --- | --- | --- | --- |
|  | | **Physical activity guideline adherence^c^** | **Overall APR**  **(95% CI)** | **Males APR**  **(95% CI)** | **Females APR**  **(95% CI)** |
| **Adverse cardiovascular conditions** | |  |  |  |  |
|  | Hypertension | Meet neither | 1 | 1 | 1 |
|  |  | MSE only | 0.74 (0.72-0.75) | 0.94 (0.92-0.96) | 0.82 (0.81-0.83) |
|  |  | MVPA only | 0.88 (0.87-0.89) | 0.71 (0.69-0.73) | 0.75 (0.72-0.77) |
|  |  | Meet both | 0.67 (0.66-0.68) | 0.75 (0.73-0.77) | 0.59 (0.58-0.61) |
|  | High cholesterol | Meet neither | 1 | 1 | 1 |
|  |  | MSE only | 0.78 (0.76-0.79) | 1.00 (0.98-1.05) | 0.89 (0.87-0.91) |
|  |  | MVPA only | 0.94 (0.93-0.95) | 0.73 (0.70-0.75) | 0.82 (0.79-0.84) |
|  |  | Meet both | 0.76 (0.75-0.78) | 0.81 (0.50-0.80) | 0.71 (0.69-0.72) |
|  | Diabetes | Meet neither | 1 | 1 | 1 |
|  |  | MSE only | 0.61 (0.59-0.63) | 0.80 (0.78-0.83) | 0.61 (0.59-0.63) |
|  |  | MVPA only | 0.69 (0.68-0.71) | 0.54 (0.51-0.58) | 0.64 (0.61-0.68) |
|  |  | Meet both | 0.44 (0.43-0.45) | 0.50 (0.48-0.52) | 0.44 (0.43-0.46) |
|  | Myocardial infarction | Meet neither | 1 | 1 | 1 |
|  |  | MSE only | 0.70 (0.66-0.74) | 0.85 (0.81-0.89) | 0.64 (0.60-0.67) |
|  |  | MVPA only | 0.76 (0.74-0.79) | 0.62 (0.57-0.66) | 0.70 (0.63-0.77) |
|  |  | Meet both | 0.54 (0.51-0.56) | 0.57 (0.54-0.60) | 0.44 (0.41-0.48) |
|  | Coronary heart disease | Meet neither | 1 | 1 | 1 |
|  |  | MSE only | 0.74 (0.70-0.78) | 0.89 (0.85-093) | 0.65 (0.62-0.69) |
|  |  | MVPA only | 0.78 (0.76-0.80) | 0.63 (0.58-0.68) | 0.79 (0.72-0.86) |
|  |  | Meet both | 0.60 (0.57-0.62) | 0.68 (0.65-0.72) | 0.44 (0.41-0.47) |
|  | Stroke | Meet neither | 1 | 1 | 1 |
|  |  | MSE only | 0.79 (0.75-0.84) | 0.76 (0.71-0.81) | 0.62 (0.58-0.65) |
|  |  | MVPA only | 0.66 (0.64-0.68) | 0.76 (0.69-0.84) | 0.82 (0.75-0.90) |
|  |  | Meet both | 0.53 (0.50-0.55) | 0.61 (0.57-0.66) | 0.47 (0.44-0.51) |
| **General adverse health conditions** | |  |  |  |  |
|  | Depressive disorder | Meet neither | 1 | 1 | 1 |
|  |  | MSE only | 0.83 (0.80-0.85) | 0.73 (0.70-0.75) | 0.73 (0.72-0.76) |
|  |  | MVPA only | 0.72 (0.71-0.73) | 0.80 (0.76-0.84) | 0.88 (0.85-0.92) |
|  |  | Meet both | 0.59 (0.58-0.61) | 0.61 (0.59-0.64) | 0.61 (0.59-0.63) |
|  | Chronic obstructive pulmonary disease | Meet neither | 1 | 1 | 1 |
|  |  | MSE only | 0.65 (0.62-0.68) | 0.55 (0.51-0.60) | 0.74 (0.69-0.78) |
|  |  | MVPA only | 0.58 (0.56-0.59) | 0.61 (0.58-0.64) | 0.55 (0.53-0.58) |
|  |  | Meet both | 0.39 (0.38-0.40) | 0.42 (0.39-0.44) | 0.37 (0.35-0.40) |
|  | Asthma | Meet neither | 1 | 1 | 1 |
|  |  | MSE only | 0.96 (0.93-0.99) | 0.99 (0.93-1.05) | 0.78 (0.75-0.81) |
|  |  | MVPA only | 0.81 (0.79-0.82) | 0.86 (0.82-0.89) | 0.80 (0.76-0.81) |
|  |  | Meet both | 0.81 (0.79-0.83) | 0.91 (0.87-0.95) | 0.78 (0.75-0.81) |
|  | Kidney disease | Meet neither | 1 | 1 | 1 |
|  |  | MSE only | 0.72 (0.68-0.77) | 0.62 (0.55-0.69) | 0.83 (0.75-0.91) |
|  |  | MVPA only | 0.66 (0.63-0.69) | 0.71 (0.66-0.76) | 0.63 (0.59-0.67) |
|  |  | Meet both | 0.51 (0.48-0.54) | 0.52 (0.48-0.57) | 0.51 (0.46-0.54) |
|  | Cancer (non-skin) | Meet neither | 1 | 1 | 1 |
|  |  | MSE only | 0.79 (0.75-0.82) | 0.72 (0.67-0.77) | 0.87 (0.82-0.92) |
|  |  | MVPA only | 0.97 (0.95-0.99) | 1.08 (1.04-1.13) | 0.93 (0.90-0.96) |
|  |  | Meet both | 0.80 (0.78-0.83) | 0.89 (0.84-0.93) | 0.78 (0.75-0.82) |
|  | Arthritis | Meet neither | 1 | 1 | 1 |
|  |  | MSE only | 0.80 (0.78-0.82) | 0.71 (0.68-0.74) | 0.91 (0.88-0.94) |
|  |  | MVPA only | 0.87 (0.85-0.88) | 0.93 (0.90-0.95) | 0.84 (0.83-0.86) |
|  |  | Meet both | 0.72 (0.70-0.73) | 0.76 (0.74-0.78) | 0.71 (0.69-0.73) |
| **Total number of adverse health conditions** | |  |  |  |  |
|  | ≥4^d^ | Meet neither | 1 | 1 | 1 |
|  |  | MSE only | 0.61 (0.58-0.64) | 0.57 (0.53-0.62) | 0.66 (0.62-0.70) |
|  |  | MVPA only | 0.74 (0.72-0.76) | 0.84 (0.57-0.64) | 0.68 (0.65-0.70) |
|  |  | Meet both | 0.50 (0.48-0.51) | 0.57 (0.54-0.60) | 0.41 (0.40-0.43) |
|  | ≥5^e^ | Meet neither | 1 | 1 | 1 |
|  |  | MSE only | 0.63 (0.59-0.67) | 0.59 (0.55-0.63) | 0.75 (0.71-0.79) |
|  |  | MVPA only | 0.63 (0.60-0.65) | 0.67 (0.65-0.70) | 0.52 (0.50-0.53) |
|  |  | Meet both | 0.42 (0.40-0.44) | 0.45 (0.43-0.48) | 0.34 (0.32-0.36) |
|  | ≥6^f^ | Meet neither | 1 | 1 | 1 |
|  |  | MSE only | 0.67 (0.63-0.71) | 0.58 (0.52-0.64) | 0.78 (0.72-0.84) |
|  |  | MVPA only | 0.50 (0.48-0.53) | 0.59 (0.56-0.63) | 0.45 (0.43-0.48) |
|  |  | Meet both | 0.33 (0.31-0.35) | 0.38 (0.35-0.41) | 0.30 (0.27-0.32) |
| ^a^ PR adjusted for sex (in overall model only), age, race/ethnicity, employment, education, income, smoking and BMI.  ^b^ to be classified as having an adverse health condition a respondent had to report having a “d*octor, nurse or other health professional*” diagnose each condition.  ^c^ categories of physical activity guideline adherence : ‘Meet neither’: MVPA= 0-149 MSE= 0-1 sessions/week; ‘MVPA only’ MVPA= ≥150 minutes/week ‘MSE only’; MSE= ≥2 sessions/week & MVPA= 0-149 minutes/week); & MSE= 0-1 sessions/week; and ‘Meet both’: MVPA= ≥150 minutes /week & MSE= ≥2 sessions/week.  ^d^ reference = ≤3 adverse conditions.  ^e^ reference = ≤4 adverse conditions.  ^f^ reference = ≤5 adverse conditions. | | | | | |
